# Dipole-allowed direct band gap silicon superlattices

**DOI:** 10.1038/srep18086

**Published:** 2015-12-11

**Authors:** Young Jun Oh, In-Ho Lee, Sunghyun Kim, Jooyoung Lee, Kee Joo Chang

**Affiliations:** 1 Department of Physics, Korea Advanced Institute of Science and Technology, Daejeon 34141, Korea; 2 Korea Research Institute of Standards and Science, Daejeon 34113, Korea; 3 Center for In Silico Protein Science, School of Computational Science, Korea Institute for Advanced Study, Seoul 02455, Korea

## Abstract

Silicon is the most popular material used in electronic devices. However, its poor optical properties owing to its indirect band gap nature limit its usage in optoelectronic devices. Here we present the discovery of super-stable pure-silicon superlattice structures that can serve as promising materials for solar cell applications and can lead to the realization of pure Si-based optoelectronic devices. The structures are almost identical to that of bulk Si except that defective layers are intercalated in the diamond lattice. The superlattices exhibit dipole-allowed direct band gaps as well as indirect band gaps, providing ideal conditions for the investigation of a direct-to-indirect band gap transition. The fact that almost all structural portions of the superlattices originate from bulk Si warrants their stability and good lattice matching with bulk Si. Through first-principles molecular dynamics simulations, we confirmed their thermal stability and propose a possible method to synthesize the defective layer through wafer bonding.

Silicon is an important element used in modern electronic devices owing to its abundance, feasibility for large-scale fabrication, easy formation of native oxide, and doping controllability of both electrons and holes. However, the optical property of Si is rather poor owing to its indirect electronic band gap nature. In an indirect-band-gap material like cubic-diamond Si (denoted as *c*-Si), optical transitions at the threshold energy occur only via momentum-conserving phonons. Therefore, the solar spectrum pertaining to the energy below the direct band gap of *c*-Si, *i.e.*, approximately 3.4 eV, cannot be effectively absorbed without phonon assistance.

To improve the optical property of Si, considerable efforts have been made; for instance, by introducing defects, such as erbium atoms, dislocations, and grain boundaries as recombination centers^1^,^2^,^3^, and/or by engineering the electronic band structure through nanopatterning^4^,^5^, nanostructuring^6^, alloying with group-IV elements^7^,^8^, applying strain, or combinations of these^9^,^10^,^11^,^12^. Very recently, several Si crystals with direct and quasidirect band gaps were computationally designed^13^,^14^,^15^ and their optical absorption properties were shown to be significantly improved compared with *c*-Si. However, these metastable structures were of relatively high energies, ranging 0.1–0.3 eV per atom, because of distorted tetrahedral bonds, and the experimental realization of their synthesis has yet to be explored.

In this work, we report low-energy pure Si-based superlattices that exhibit direct and optically allowed band gaps with good lattice matching with *c*-Si. The superlattice structure is composed of alternating stacks of bulk-like Si(111) layers and a defective layer containing Seiwatz chains^16^. The electronic structure evolves as the bulk-like Si portion increases, exhibiting a transition from the direct to indirect band gap. In superlattices with direct band gaps, the optical transition at the threshold energy is greatly enhanced. Hence we suggest that these superlattices could be used in thin-film solar cells. Based on molecular dynamics simulations, we discuss the thermal stability and the possible synthesis through wafer bonding.

## Results and Discussion

### Calculation method

We explored Si crystal structures with optically active direct band gaps by using the computational search method, in which electronic properties are initially assigned and target materials are subsequently searched^17^. We employed a combined approach^13^ of conformational space annealing (CSA) for global optimization^18^,^19^,^20^,^21^ and first-principles calculations within the density functional theory (DFT) framework^22^,^23^ for enthalpy evaluation. We optimized the degrees of freedom, including atomic positions 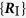 and six lattice parameters (*a*, *b*, *c*, *α*, *β*, and *γ*) without using any specific knowledge of known Si crystals. The objective function used for selection in CSA is 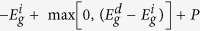, where 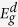 and 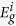 denote direct and indirect band gaps, respectively. The penalty function 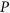 was set to be 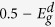, 0, and 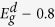 for 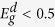, 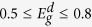, and 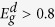, respectively, so that the objective function promotes the formation of direct band gaps in the range of 0.5–0.8 eV (which will be discussed shortly).

For each conformation, the enthalpy was minimized by performing DFT calculations which used the functional form of Perdew, Burke, and Ernzerhof (PBE) for the exchange-correlation potential^24^ and the projector augmented wave potentials^25^, as implemented in the VASP code^26^. The wave functions were expanded in plane waves with the energy cutoff of 400 eV. With a **k**-point mesh using the grid spacing of 2π × 0.02 Å^−1^, the crystal structures were minimized until all forces and stress tensors were less than 0.01 eV Å^−1^ and 1.5 kbar, respectively. We tested the minimization convergence using the higher energy cutoff of 600 eV and the smaller minimization parameter values of 0.001 eV Å^−1^ and 0.07 kbar for force and stress, respectively, and found that the enthalpy and the PBE band gap were accurate within about 0.02 meV/atom and 3 meV, respectively. Finally, a twice finer **k**-point mesh was used to determine the nature of band gap.

The band gap sizes of semiconductors and insulators are usually underestimated with the PBE functional (by about 47% for *c*-Si). The ideal band gap size for solar cell applications is 1.1–1.3 eV^27^, which is approximately translated to 0.6–0.7 eV by PBE. Thus, the target PBE direct band gaps with the penalty function lie in the range of 0.5–0.8 eV. For more accurate calculations, we additionally performed quasiparticle calculations in the *G*_0_*W*_0_ approximation^28^,^29^ and solved the Bethe-Salpeter equation^30^, considering up to 12 occupied and 16 unoccupied bands around the Fermi level, which were shown to be sufficient to ensure the numerical convergence^13^.

### Silicon superlattice structures

**Figure 1:**
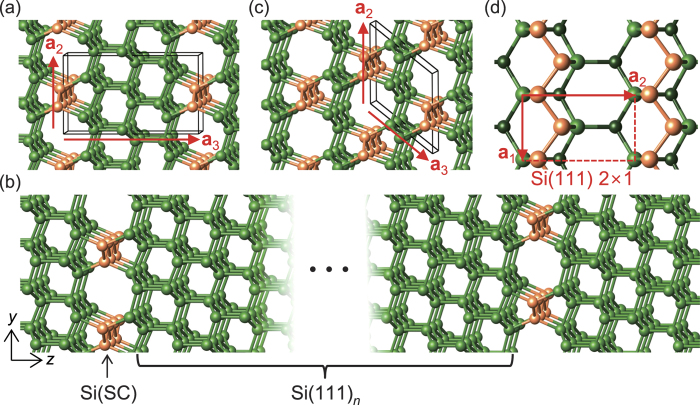
The Si(111)_*n*_/Si(SC) superlattices composed of Si(111) layers (green) and a defective layer of Seiwatz chains (orange) are shown for (**a**) *n* = 3, (**b**) arbitrary *n*, and (**c**) *n* = 1. The black parallelepiped represents the unit cell spanned by the lattice vectors, ***a***_1_, ***a***_2_, and ***a***_3_. (**d**) The orientation of Seiwatz chains on the Si(111) 2 × 1 surface is shown.

After an extensive search for Si crystal structures with optically active direct band gaps, we obtained a very distinctive superlattice structure, especially for the system containing 14 Si atoms per unit cell. While the previously reported Si allotropes^13^,^14^,^15^ are centeracterized by severely distorted tetrahedral bonds, the superlattice structure consists of alternating stacks of three Si(111) layers and a defective layer along the [111] direction of *c*-Si (Fig. 1a). The defective layer contains the so-called Seiwatz chains (SCs)^16^, which were suggested to be formed via 2 × 1 reconstruction on the Si(111) surface with half-monolayer coverage. The SCs lead to open channels consisting of five- and eight-membered rings in the defective region. Note that all Si atoms are four-fold coordinated, without any coordination defects, and nearly ideal tetrahedral bonds are formed in the non-defective region. Owing to the almost identical structural match between the Si(111) layers and *c*-Si, many additional superlattice structures [denoted as Si(111)_*n*_/Si(SC)] can be constructed by varying *n*, as shown in Fig. 1b. For *n* = 1, the crystal is entirely composed of five- and eight-membered rings (Fig. 1c), very similar to an orthorhombic allotrope, Si_24_, which has been recently synthesized^31^.

In the cubic diamond (Si-I) phase, the stacking sequence of the Si(111) layer is *ABCABC*…, while that of the hexagonal diamond (lonsdaleite, Si-IV) phase is *ABABAB*…. In our superlattice systems, various stacking sequences of the Si(111) layers, including the cubic and hexagonal ones, are possible, as in SiC polytypes. In addition, in each defective layer, two configurations of SCs exist, which are related to each other by the translation of 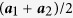, where 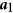 and 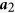 are the lattice vectors on the 2 × 1 basal plane (Fig. 1d). Thus, depending on the relative positions of two adjacent defective layers, two Bravais lattices can be formed, simple monoclinic (SM) and base-centered monoclinic (BCM) for *n* ≥ 2, with some exceptions for the case of hexagonal-diamond stacking (Table 1). By examining various superlattices, we found that BCM superlattices tend to yield quasidirect/indirect band gaps, whereas the nature of the band gaps (direct vs quasidirect) in the SM ones varies with *n*. Here quasidirect band gaps are defined as 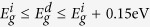, following the previous study^13^.

### Direct-to-indirect band gap transition

**Figure 2:**
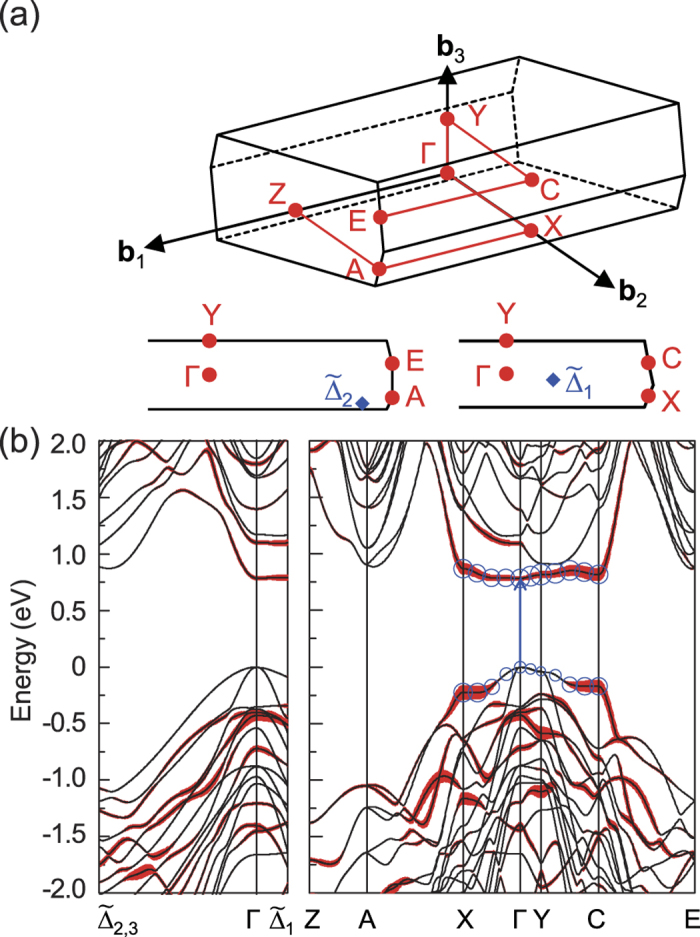
(**a**) Symmetry points and lines in the Brillouin zone of the simple monoclinic lattice are shown. (**b**) The PBE band structure of the Si(111)_*n* = 5_/Si(SC) superlattice is shown. The thickness of red colored bands represents the degree of confinement in the defective layer, indicating that the lowest conduction band along the X-Γ-Y-C line is mainly derived from the Seiwatz chains. For the highest valence and lowest conduction bands along the X-Γ-Y-C line, the size of blue circles is proportional to the degree of contribution from the interface layer.

**Figure 3:**
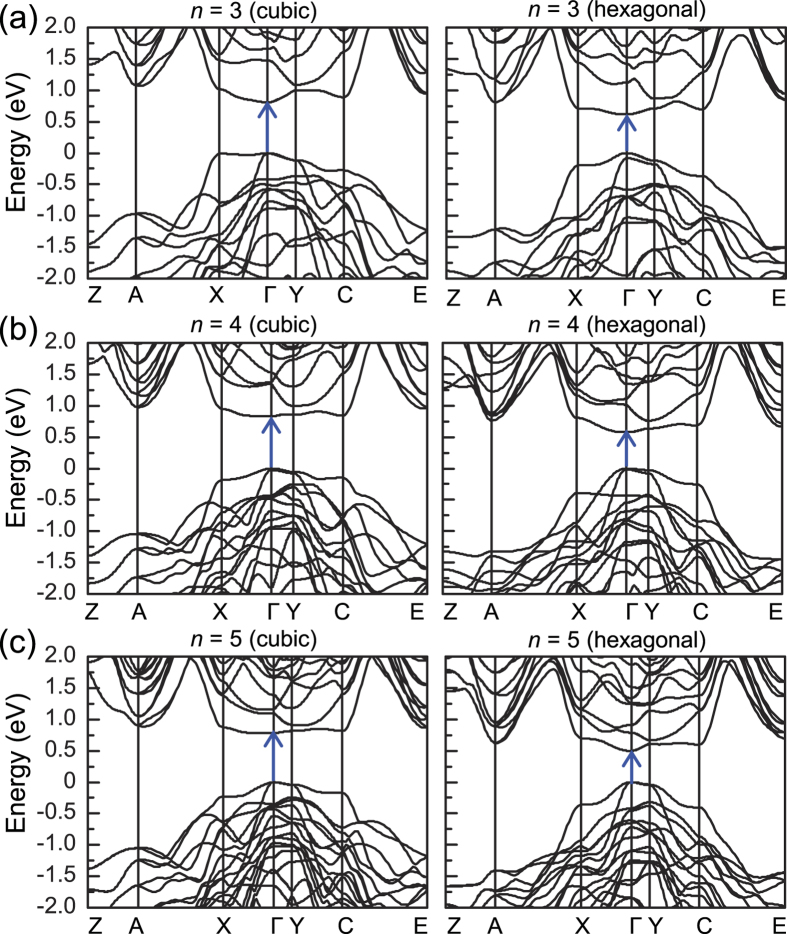
PBE band structures of the Si(111)_*n*_/Si(SC) superlattices with the cubic- and hexagonal-diamond stacking sequences of the Si(111) layers are shown for (a) *n* = 3, (b) *n* = 4, and (c) *n* = 5.

Because *c*-Si is the most stable form of Si, we focused on SM superlattices with cubic-diamond stacking; throughout this paper, unless otherwise specified, the cubic stacking is considered. For superlattices with *n* up to *n* = 13, the valence band maximum (VBM) is always located at the Γ point, the center of the Brillouin zone (BZ). For *n* = 3–5, we found direct band gaps at the Γ point (Table 1 and Figs 2 and 3), which were estimated to be 0.807, 0.832, and 0.782 eV, respectively, using the PBE exchange-correlation functional. As *n* increased, a direct-to-quasidirect band gap transition occurred. The energy differences between the direct and indirect band gaps were smaller than 30 meV for 6 ≤ *n* ≤ 10. Although the band gap size decreased with increasing *n*, it was larger than that (0.62 eV) of *c*-Si, owing to the quantum confinement effect. When more rigorous quasiparticle *G*_0_*W*_0_ calculations were performed, the nature of the band gap did not change. The *G*_0_*W*_0_ band gaps were 1.15–1.28 eV (*n* = 3–10), close to the optimal value (1.1–1.3 eV) for solar cell applications^27^ (Table 1). For comparison, we also examined SM superlattices with hexagonal-diamond stacking and found direct band gaps at the Γ point for *n* = 3–5. However, the band gap sizes were reduced by approximately 0.19–0.29 eV (Table 1), similar to SiC polytypes, where the band gap tends to decrease as the number of hexagonal layers in stacking sequence increases.

**Figure 4:**
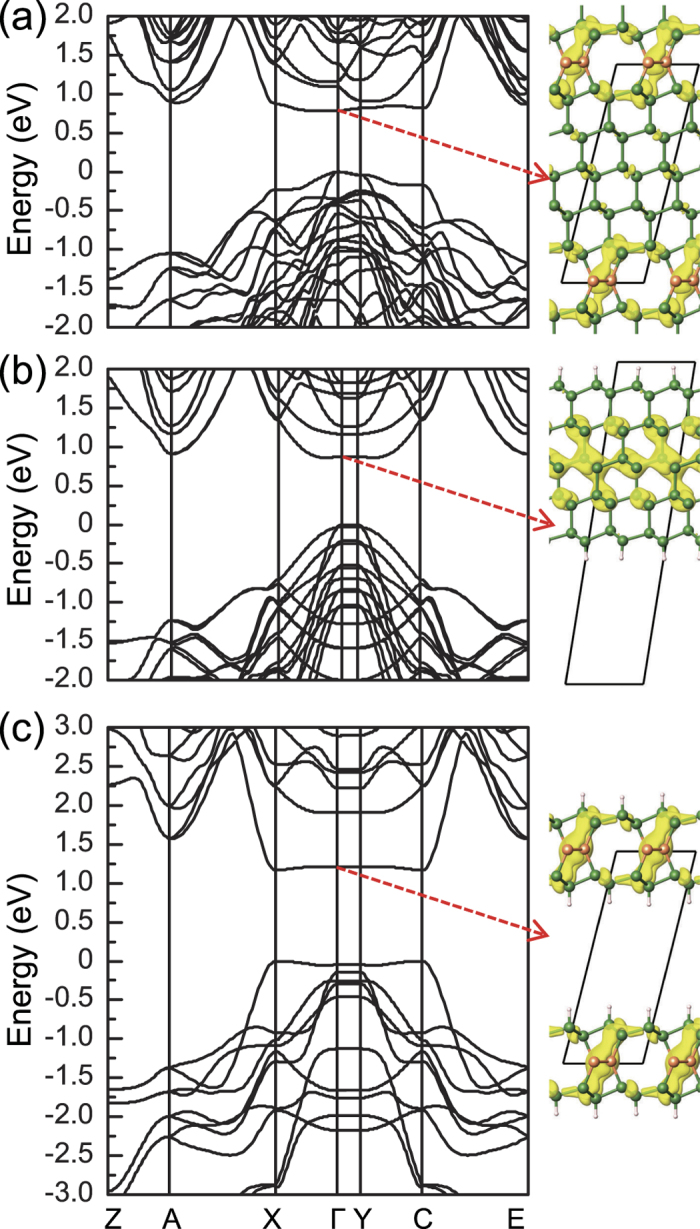
The PBE band structures (left panel) are compared for (a) the Si(111)_*n* = 5_/Si(SC) superlattice with the cubic-diamond stacking sequence of the Si(111) layers, (b) a slab geometry consisting of five Si(111) layers and a vacuum region, and (c) a slab geometry consisting of a defective layer sandwiched between two Si(111) layers and a vacuum region. Surface Si dangling bonds are passivated by hydrogen. In right panel, isosurfaces (yellow) of the centerge densities of the lowest conduction bands at the Γ point are drawn.

**Figure 5:**
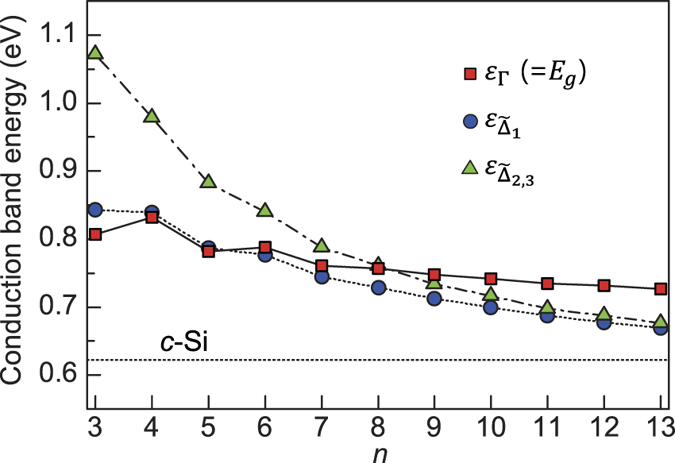
Direct-to-indirect band gap transition of the Si(111)_*n*_/Si(SC) superlattices. The lowest conduction band energies at the Γ, 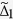, and 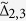 points are plotted as a function of *n* and compared with that of *c*-Si (horizontal dotted line).

In our superlattice system, the lowest conduction band exhibits nearly flat dispersion along the Γ-X and Γ-Y directions (Figs 2 and 3). To understand the origin of the flat dispersion of the lowest conduction band around the BZ center, we considered the defective and Si(111) layers separately (Fig. 4). In a slab geometry with a single defective layer sandwiched between two Si(111) layers, a vacuum region was included in the same unit cell and Si dangling bonds were passivated by hydrogen. The flat dispersion occurs along the directions perpendicular to the alignment of the SCs, while the dispersion becomes significant to the alignment direction. Thus, the flat band is attributed to the SCs in the defective layer. On the other hand, the energy states derived from the Si(111) layers are affected by the zone folding and quantum confinement effects. In *c*-Si, the conduction band minimum (CBM) is located at six Δ-valleys close to the X points in the BZ of the face-centered cubic lattice. When a SM cell with a 2 × 1 basal plane is adopted, one of the three X points is folded to the BZ center in the monoclinic BZ (Supplementary Methods and Supplementary Fig. 1). In contrast to *c*-Si, the hexagonal symmetry in the superlattice is perturbed by the SCs (Fig. 1d); hence, six Δ-valley states split into two folded states, 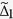 and 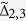, which have two- and four-fold degeneracies, respectively. The folded 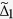 points are located near the Γ point, whereas the folded 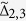 points are close to the A or E point in the BZ, depending on *n* (Fig. 2a). The lowest conduction bands at the Γ, 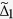, and 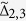 points are shown as a function of *n* in Fig. 5. While the 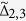 states are mainly confined in the Si(111) layers, reflecting their zone folding nature, the centeracteristics of 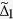 vary with *n*.

**Figure 6:**
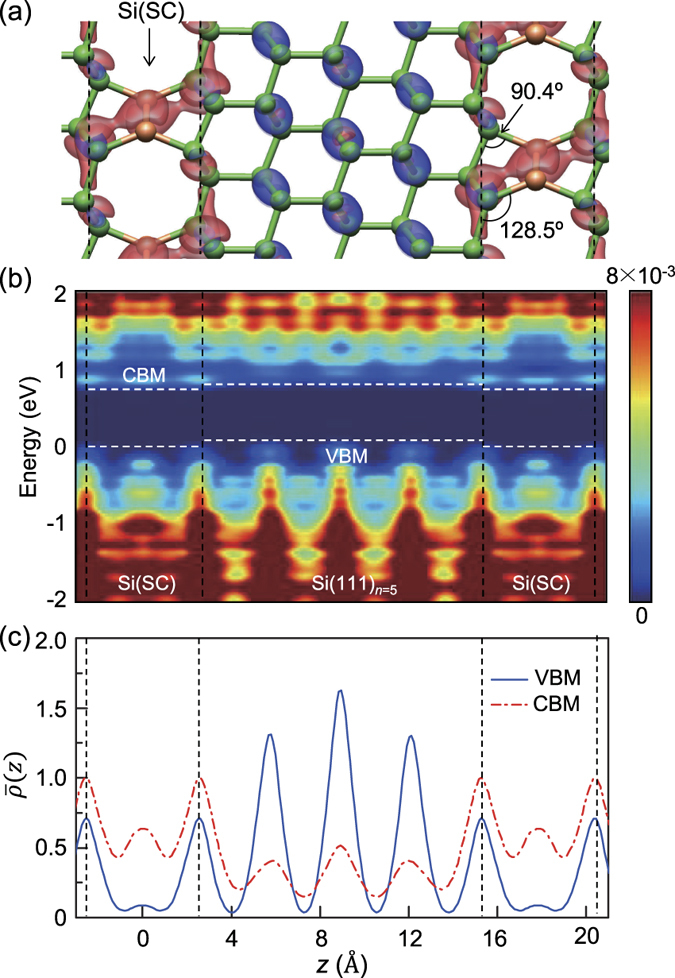
For the Si(111)_*n* = 5_/Si(SC) superlattice, (a) isosurfaces (1.32 × 10^‒2^ electrons Å^‒3^) of the centerge densities of VBM (blue) and CBM (red), (b) local density of states per unit volume (in units of electrons/eV) averaged over the *xy* plane, and (c) planar-averaged centerge densities per unit volume (in units of 10^‒2^ electrons) of VBM and CBM are plotted along the superlattice direction (*z*-axis). Black dotted lines denote the position of interface Si(111) layers and white dotted lines represent the approximate positions of the band edge states in the middle of the defective and non-defective regions.

The nature of the band gap in our superlattices was determined by the competition between the novel defect-derived flat state and the folded states of the Si(111) layers in the conduction band. For small *n*, the band gap of the non-defective region becomes large due to the quantum confinement effect. Thus, the defect-derived flat band at the Γ point is lower than all the folded states, exhibiting a direct band gap behaviour up to *n* = 5. The planar-averaged centerge densities clearly show that CBM is largely confined in the defective region, while VBM is mainly derived from the Si(111) layers (Fig. 6). For large *n*, as the confinement effect is reduced, the folded 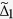 states move down below the defect-derived flat band, resulting in a direct-to-quasidirect band gap transition around *n* = 6 (Fig. 5).

### Optical transition and photovoltaic efficiency

**Figure 7:**
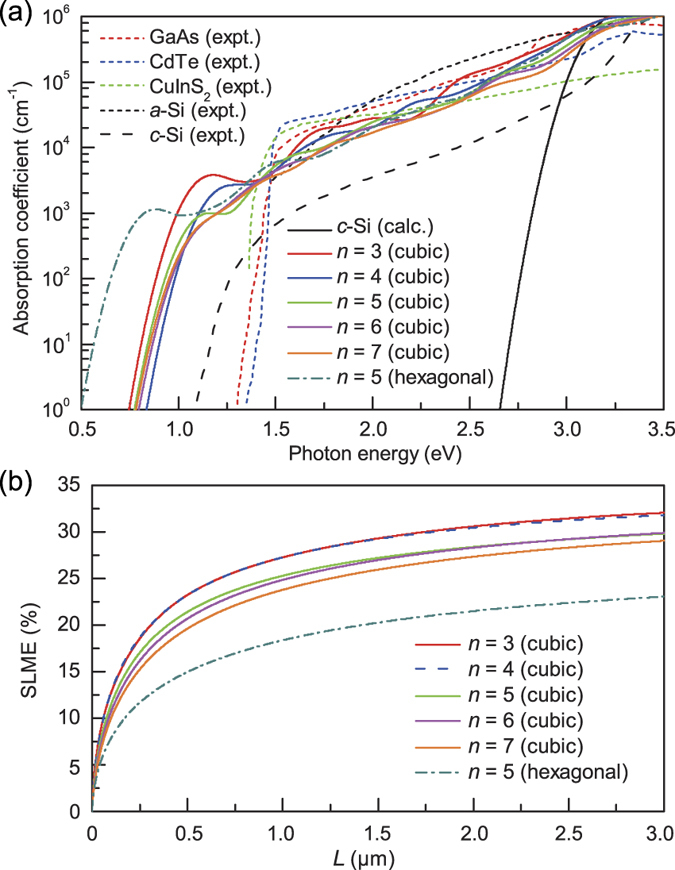
For various Si(111)_*n*_/Si(SC) superlattices with the cubic- and hexagonal-diamond stacking sequences of the Si(111) layers, (a) the calculated absorption coefficients are compared with the experimentally measured values (from refs 32,33,34,35) for GaAs, CdTe, CuInS_2_, amorphous Si (*a*-Si), and *c*-Si and (b) the spectroscopic limited maximum efficiency (SLME) is plotted as a function of film thickness *L*.

The novel defect-derived band plays an important role in strong optical transitions near the threshold energy. The squares of the dipole matrix elements, 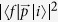, for the direct transition at the Γ point were calculated to be 0.173, 0.141, and 0.045 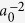 in atomic units for *n* = 3–5, respectively, where 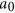 is the Bohr radius. These values are higher than 0.03 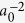, obtained from the dipole-allowed direct band gap of a specially designed Si/Ge superstructure^11^, indicating that the optical transition was greatly enhanced in our case. Such strong dipole-allowed transitions are partly attributed to the large overlap of the band edge states at the interface layers (Figs 2b and 6). In addition, the hybridization of the conduction band states is another critical factor for the dipole-allowed transition. Because of the distorted tetrahedral bonds around the interface Si atoms, the *p* orbital centeracter is significantly enhanced for the CBM state (Supplementary Fig. 2), as in the dipole-allowed transition at the direct gap of *c*-Si. The calculated absorption coefficients of our superlattices are comparable to those of direct band gap semiconductors, such as GaAs, CdTe, and CuInS_2_ (Fig. 7a), which are known as good photovoltaic materials. The spectroscopic limited maximum efficiency^36^ for the sample thickness of *L* = 2.0 μm was estimated to be in the range of 27%–31% for *n* = 3–7 (Fig. 7b), indicating that the optical absorption properties were excellent even for the quasidirect band gap superlattices.

**Figure 8:**
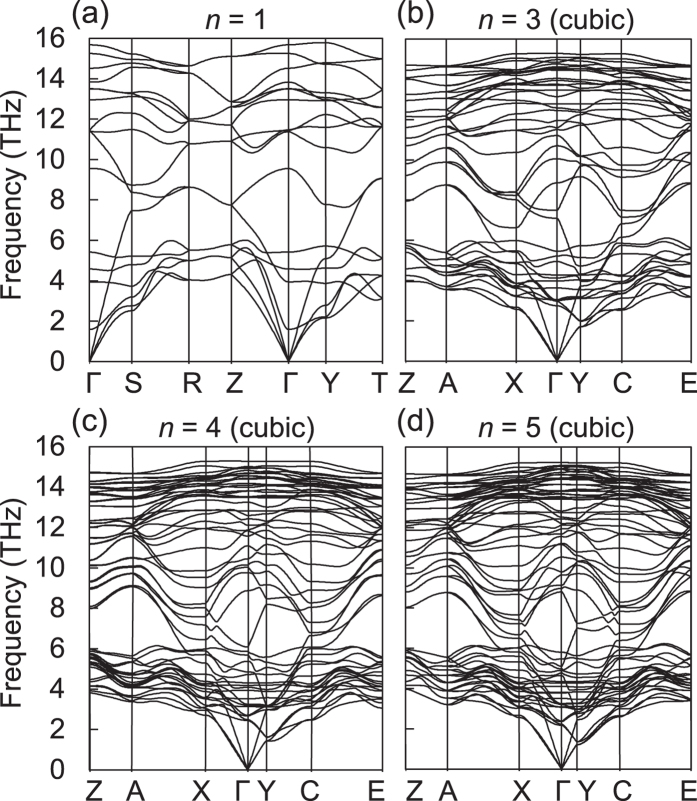
For the Si(111)_*n*_/Si(SC) superlattices with the cubic-diamond stacking sequence of the Si(111) layers, the calculated phonon spectra are shown for *n* = 1, 3, 4, and 5. The 2 × 2 × 3, 3 × 2 × 2, 3 × 2 × 2, and 3 × 2 × 2 supercells were chosen for *n* = 1, 3, 4, and 5, respectively.

**Figure 9:**
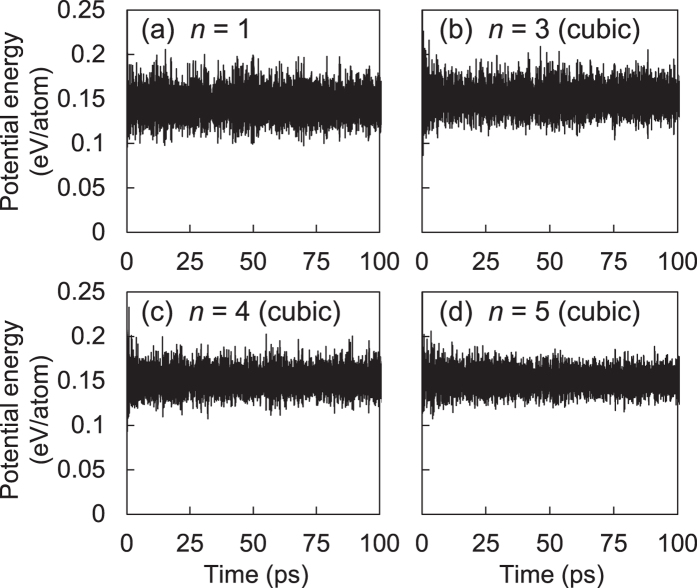
For the Si(111)_*n*_/Si(SC) superlattices with the cubic-diamond stacking sequence of the Si(111) layers, the thermal stability was examined by performing first-principles MD simulations at 1100 K, with choosing the 2 × 2 × 3, 3 × 2 × 1, 3 × 2 × 1, and 3 × 2 × 1 supercells for *n* = 1, 3, 4, and 5, respectively.

Note that our superlattices are both energetically and dynamically stable at the ambient condition. Since the Si(111) layers are structurally almost identical to *c*-Si, their excess energies are quite low, ranging 0.013–0.042 eV per atom for *n* = 3–10 (Table 1). These energies are lower by an order of magnitude than those (0.1–0.3 eV per atom) of the previously predicted Si allotropes with direct and quasidirect band gaps^13^,^14^,^15^. For our direct band gap superlattices, we found no imaginary phonon modes in the phonon spectra (Fig. 8). In addition, we examined their thermal stability by performing first-principles molecular dynamics (MD) simulations and confirmed that they were thermally stable up to 100 ps at the high temperature of 1100 K (Fig. 9). Although 100 picoseconds MD simulations may not be long enough to warrant the long-term stability, our simulation time is about 10 times longer than usual first-principles MD simulation times^14^,^15^. In addition, since the simulation was performed at 1100 K, it is not outrageous to assume that the superlattice structure would be long-term stable at room temperature.

### Possible synthesis route

**Figure 10:**
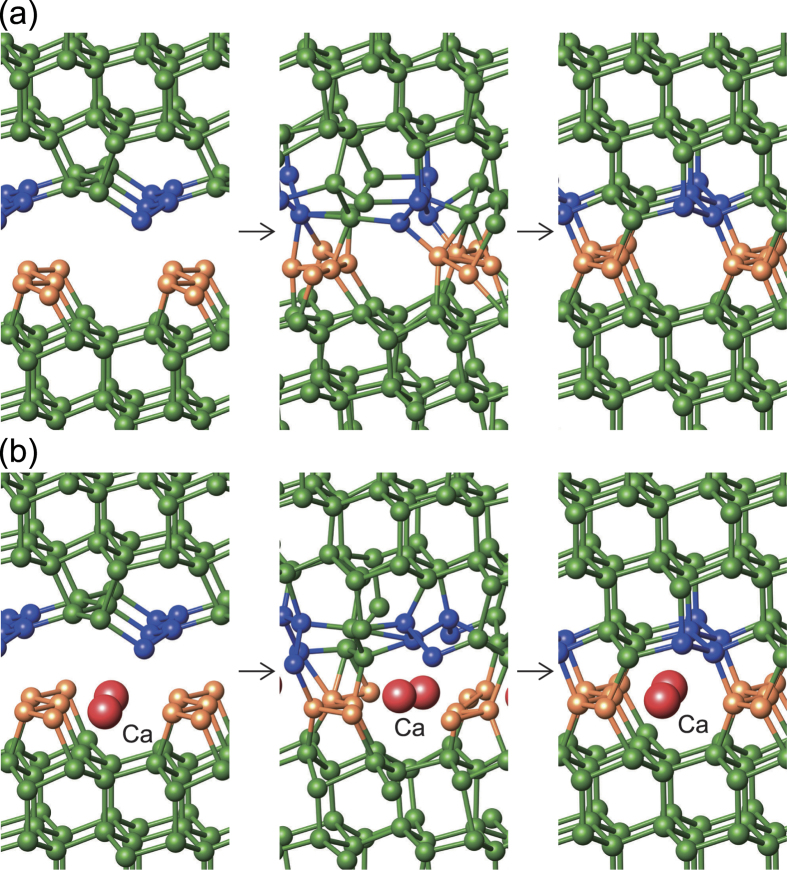
Molecular dynamics simulations for wafer bonding. (**a**) For the initial configuration where one surface has Pandey π-bonded chains (blue circles) and the other has Seiwatz chains (orange circles), an intermediate configuration after 2 ps and the final configuration after 3 ps at 1100 K are shown. (**b**) For the initial configuration where Ca atoms are adsorbed between Seiwatz chains on one surface, an intermediate configuration after 3 ps and the final configuration after 7 ps at 1100 K are shown. In the final state, the Ca atoms are aligned along the open channels of eight-membered rings.

We examined the possibility of creating a defective layer containing SCs, which can lead to the eventual realization of our superlattice structures. We performed first-principles MD simulations for wafer bonding between two Si(111) 2 × 1 surfaces. For the initial configuration, we prepared a 4 × 2 lateral supercell, where one surface had Pandey π-bonded chains^37^ and the other SCs^16^. A vacuum region of 15 Å thickness was inserted between the other two surfaces passivated by hydrogen. We found that a defective layer with the SCs was formed after about 3 ps at 1100 K (Fig. 10a). In the process of wafer bonding, the formation of a defective layer with SCs is accompanied with bond breaking and re-bonding between the two Si surfaces. Thus, this process is unlikely to take place at much lower temperatures than 1100 K. We note that, at 900 K , the wafer bonding was not completed within the observed time span of about 13 s.

In the above wafer bonding setup, the 2 × 1 reconstruction of the SCs may be difficult to prepare, because it is metastable with respect to the Pandey reconstruction model. On the other hand, divalent adsorbates, such as Ca, Sr, and Ba, can stabilize the SCs at half-monolayer coverage^38^,^39^. Thus, we considered one of the 2 × 1 surfaces with Ca atoms adsorbed at hollow surface sites (Fig. 10b) and repeated the wafer bonding simulation. We found that the same defective layer was formed after approximately 7 ps. In the final configuration, the Ca adsorbates resided along the open channels of eight-membered rings, as in the case of the Eu_4_Ga_8_Ge_16_-type structure found in CaSi_6_, SrSi_6_, BaSi_6_^40^, and recently in NaSi_6_^31^,^41^. Once the defective layer is formed, the adsorbed Ca atoms can be removed via a diffusion process along the channel, as experimentally observed in the case of NaSi_6_^31^. Thus, the wafer bonding between the clean and divalent-adsorbed Si(111) surfaces can serve as a promising technique for the synthesis of superlattices containing the defective layers.

## Conclusion

In conclusion, using a computational search method, we have discovered low-energy pure-Si superlattice structures with dipole-allowed direct band gaps, which can serve as promising materials for solar cell applications. As the bulk-like Si portion increases, the superlattice system exhibits a transition from the direct to indirect band gap. The electronic evolution can be understood in terms of a novel conduction band originating from defective layers, an overlap between the valence- and conduction-band edge states at the interface layers, and zone folding with quantum confinement effects on the conduction band of non-defective bulk-like Si. The current methodology can be applied to other semiconductors to design materials with intended properties. The method proposed for the synthesis of the defective layer through wafer bonding can be followed up by experiments and could herald a new optoelectronic era.

### Computational cost

In the crystal structure search, a large amount of computational resources was used to discover the superlattice structure for the *N* = 14 system, where *N* is the number of Si atoms per unit cell. We performed the local enthalpy minimization for about 15,000 configurations, for which roughly 600 CPU cores were used for a month. We note that our superlattice structure is quite stable with its energy much lower than those of the previously reported allotropes^13^ with quasidirect and indirect band gaps for the same *N* = 14 system. Although our superlattice may be the lowest metastable allotrope for the system, one cannot prove it because the CSA approach used in the current crystal structure search is a heuristic and stochastic method.

## Additional Information

**How to cite this article**: Oh, Y. J. *et al.* Dipole-allowed direct band gap silicon superlattices. *Sci. Rep.* **5**, 18086; doi: 10.1038/srep18086 (2015).

## Supplementary Material

Supplementary Information

## Figures and Tables

**Table 1:** Summary of calculations for Si(111)_*n*_/Si(SC) superlattices.

*n*	lattice	*N*	*E*	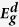 (PBE)	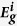 (PBE)	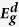 (*G*_0_*W*_0_)	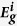 (*G*_0_*W*_0_)
1	BCO	6	89 (QD)	0.431	0.430	0.894	0.847
	Cubic-diamond stacking						
2	SM	10	46 (QD)	0.906	0.869	1.346	1.316
3	SM	14	42 (D)	0.807		1.197	
4	SM	18	32 (D)	0.832		1.283	
5	SM	22	26 (D)	0.782		1.218	
6	SM	26	22 (QD)	0.788	0.774	1.224	1.215
7	SM	30	19 (QD)	0.761	0.741	1.198	1.185
8	SM	34	17 (QD)	0.746	0.726	1.185	1.166
9	SM	38	16 (QD)	0.738	0.712	1.177	1.154
10	SM	42	13 (QD)	0.725	0.699	1.167	1.147
	Hexagonal-diamond stacking						
2	SO	10	72 (QD)	0.562	0.527	0.980	0.961
3	SM	14	49 (D)	0.615		1.049	
4	SO	18	39 (D)	0.578		1.008	
5	SM	22	34 (D)	0.497		0.914	

The lattice type, the number of atoms per unit cell (*N*), the energy relative to cubic-diamond Si (*E* in meV/atom), the type of band gap, the direct band gap size (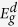 in eV), and the indirect band gap size (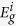 in eV) are compared for the Si(111)_*n*_/Si(SC) superlattices with the cubic- and hexagonal-stacking sequences of the Si(111) layers. The quasiparticle *G*_0_*W*_0_ gaps are also shown for comparison. Here D and QD in parentheses denote direct and quasidirect band gaps, respectively, and lattice types are abbreviated, such as SM: simple monoclinic, SO: simple orthorhombic, and BCO: base-centered orthorhombic.
