# Supplementary material for: Dipole-allowed direct band gap silicon superlattices
Source: Sci Rep. 2015 Dec 11;5:18086. doi: 10.1038/srep18086 (PMC4676021; doi:10.1038/srep18086)
Supplement: Supplementary Information [file srep18086-s1.doc]

**Supplementary Information**

Dipole-allowed direct band gap silicon superlattices

Young Jun Oh,^1^ In-Ho Lee,^2,3^ Sunghyun Kim,^1^ Jooyoung Lee^3,*^ and Kee Joo Chang^1,†^

^1^Department of Physics, Korea Advanced Institute of Science and Technology, Daejeon 305-338, Korea

^2^Korea Research Institute of Standards and Science, Daejeon 305-340, Korea

^3^Center for *In Silico* Protein Science, School of Computational Science, Korea Institute for Advanced Study, Seoul 130-722, Korea

*Corresponding author. E-mail: [jlee@kias.re.kr](mailto:jlee@kias.re.kr). Telephone number: 82-2-958-3869. Fax number: 82-2-958-3890.

^†^Corresponding author. E-mail: [kjchang@kaist.ac.kr](mailto:kjchang@kaist.ac.kr). Telephone number: 82-42-350-2531. Fax number: 82-42-350-2510.

**Supplementary Methods**

**Zone folding effect in Si(111)***_n_***/Si(SC) superlattices**

For cubic-diamond Si (*c*-Si), we obtain the optimized lattice parameter of *a* = 5.47 Å, with the PBE functional. Then, the X points in the fcc BZ (in units of 2πÅ^-1^) are written as

**X**_1_ = ( 0, -0.149, 0.106),

**X**_2_ = ( 0.129, 0.0746, 0.106),

**X**_3_ = ( -0.129, 0.0746, 0.106).

Thus, the Δ-valleys located on the Γ-X lines (84% from the zone center) are given by

**Δ**_1_ = ( 0, -0.125, 0.089),

**Δ**_2_ = ( 0.108, 0.0627, 0.089),

**Δ**_3_ = ( -0.108, 0.0627, 0.089).

The inversion of coordinates leads to the other three Δ-valleys. Here we choose the [$10\bar{1}$], [$1\bar{2}1$], and [111] directions of *c*-Si as the *x*-, *y*-, and *z*-axis, respectively.

In Si(111)*_n_*/Si(SC) superlattices, the 2×1 lateral cell is used on the basal *xy* plane. Then, for a given *n*, two Bravais lattices, simple monoclinic and base-centered monoclinic, can be formed when the cubic-diamond stacking sequence of the Si(111) layers is considered. The X points of *c*-Si are folded in the superlattice BZ.

1. **Simple monoclinic cell**

In Si(111)*_n_*/Si(SC) superlattices with *n* = 3*p* + *q*, where *p* and *q* are positive integer, *q* < 3, and *p* ≥ 1, when simple monoclinic cells are formed, the lattice vectors (in units of Å) are

**a**_1_ = ( 3.868, 0, 0 ),

**a**_2_ = ( 0, 6.699, 0 ),

**a**_3_ = ( 0, 2.233*q*, 3.158*n*+2.038 ).

Since structural relaxations depend on *n*, the actual lattice vectors are slightly deviated within 0.5%. Then, the reciprocal lattice vectors (in units of 2πÅ^-1^) become

**b**_1_ = ( 0.259, 0, 0 ),

**b**_2_ = ( 0, 0.149, -0.106*q*/(*n*+0.645) ),

**b**_3_ = ( 0, 0, 0.317/(*n*+0.645) ).

In the simple monoclinic BZ, the folded X points can be expressed in terms of the reciprocal lattice vectors as follows:

| *n* | **X**_1_ | **X**_2_ | **X**_3_ |
| --- | --- | --- | --- |
| 3 | -**b**_2_+1.22**b**_3_ | 0.5**b**_1_+0.5**b**_2_+1.22**b**_3_ | -0.5**b**_1_+0.5**b**_2_+1.22**b**_3_ |
| 4 | -**b**_2_+1.22**b**_3_ | 0.5**b**_1_+0.5**b**_2_+1.72**b**_3_ | -0.5**b**_1_+0.5**b**_2_+1.72**b**_3_ |
| 5 | -**b**_2_+1.22**b**_3_ | 0.5**b**_1_+0.5**b**_2_+2.22**b**_3_ | -0.5**b**_1_+0.5**b**_2_+2.22**b**_3_ |
| 6 | -**b**_2_+2.22**b**_3_ | 0.5**b**_1_+0.5**b**_2_+2.22**b**_3_ | -0.5**b**_1_+0.5**b**_2_+2.22**b**_3_ |
| 7 | -**b**_2_+2.22**b**_3_ | 0.5**b**_1_+0.5**b**_2_+2.72**b**_3_ | -0.5**b**_1_+0.5**b**_2_+2.72**b**_3_ |
| 8 | **-b**_2_+2.22**b**_3_ | 0.5**b**_1_+0.5**b**_2_+3.22**b**_3_ | -0.5**b**_1_+0.5**b**_2_+3.22**b**_3_ |

Note that the **X**_1_ point of *c*-Si is always folded to a **k**-point near the BZ center, regardless of *n* (*n* ≥ 3). On the other hand, the folded **X**_2_ and **X**_3_ points are close to the A (for *q* = 0, 2) or E (for *q* = 1) point in the simple monoclinic BZ (see Fig. 2a).

1. **Base-centered monoclinic cell**

In the case of base-centered monoclinic cell, the lattice vectors (in units of Å) are

**a**_1_ = ( 3.868, 0, 0 ),

**a**_2_ = ( 0, 6.699, 0 ),

**a**_3_ = ( 1.934, 3.350+2.233*q*, 3.158*n*+2.038 ).

Then, the reciprocal lattice vectors (in units of 2πÅ^-1^) become

**b**_1_ = ( 0.259, 0, -0.158/(*n*+0.645) ),

**b**_2_ = ( 0, 0.149, -(0.158+0.105*q*)/(*n*+0.645) ),

**b**_3_ = ( 0, 0, 0.315/(*n*+0.645) ).

In the base-centered monoclinic BZ, the folded X points can be expressed in terms of the reciprocal lattice vectors as follows:

| *n* | **X**_1_ | **X**_2_ | **X**_3_ |
| --- | --- | --- | --- |
| 3 | -**b**_2_+0.72**b**_3_ | 0.5**b**_1_+0.5**b**_2_+1.72**b**_3_ | -0.5**b**_1_+0.5**b**_2_+1.22**b**_3_ |
| 4 | -**b**_2_+0.72**b**_3_ | 0.5**b**_1_+0.5**b**_2_+2.22**b**_3_ | -0.5**b**_1_+0.5**b**_2_+1.72**b**_3_ |
| 5 | -**b**_2_+0.72**b**_3_ | 0.5**b**_1_+0.5**b**_2_+2.72**b**_3_ | -0.5**b**_1_+0.5**b**_2_+2.22**b**_3_ |
| 6 | -**b**_2_+1.72**b**_3_ | 0.5**b**_1_+0.5**b**_2_+2.72**b**_3_ | -0.5**b**_1_+0.5**b**_2_+2.72**b**_3_ |
| 7 | -**b**_2_+1.72**b**_3_ | 0.5**b**_1_+0.5**b**_2_+3.22**b**_3_ | -0.5**b**_1_+0.5**b**_2_+2.72**b**_3_ |
| 8 | -**b**_2_+1.72**b**_3_ | 0.5**b**_1_+0.5**b**_2_+3.72**b**_3_ | -0.5**b**_1_+0.5**b**_2_+3.22**b**_3_ |

While the folded **X**_2_ and **X**_3_ points are close to the A or E point, the **X**_1_ point is folded to a **k**-point near the Y point in the BZ of the base-centered monoclinic cell, regardless of *n* (*n* ≥ 3).

**Orbital characteristics of the lowest conduction band**

In *c*-Si, the band edge states at the Γ point are characterized by bonding and antibonding *p* orbitals and the optical transition at the direct gap is dipole-allowed. In our superlattices with direct band gaps, we examined the orbital characteristics by projecting the wave function onto the atom-centered spherical harmonics within a sphere of 1.5 Å radius. In flanking Si(111) layers, tetrahedral bonds, especially around the interface Si atoms bonded to the SCs, are distorted from their ideal values, with the bond angles of 90.4° and 128.5° (see Supplementary Figure 2). As a consequence, the *p* orbital character is significantly enhanced around one of the interface Si atoms that form the five-membered ring, leading to the strong dipole-allowed transition.


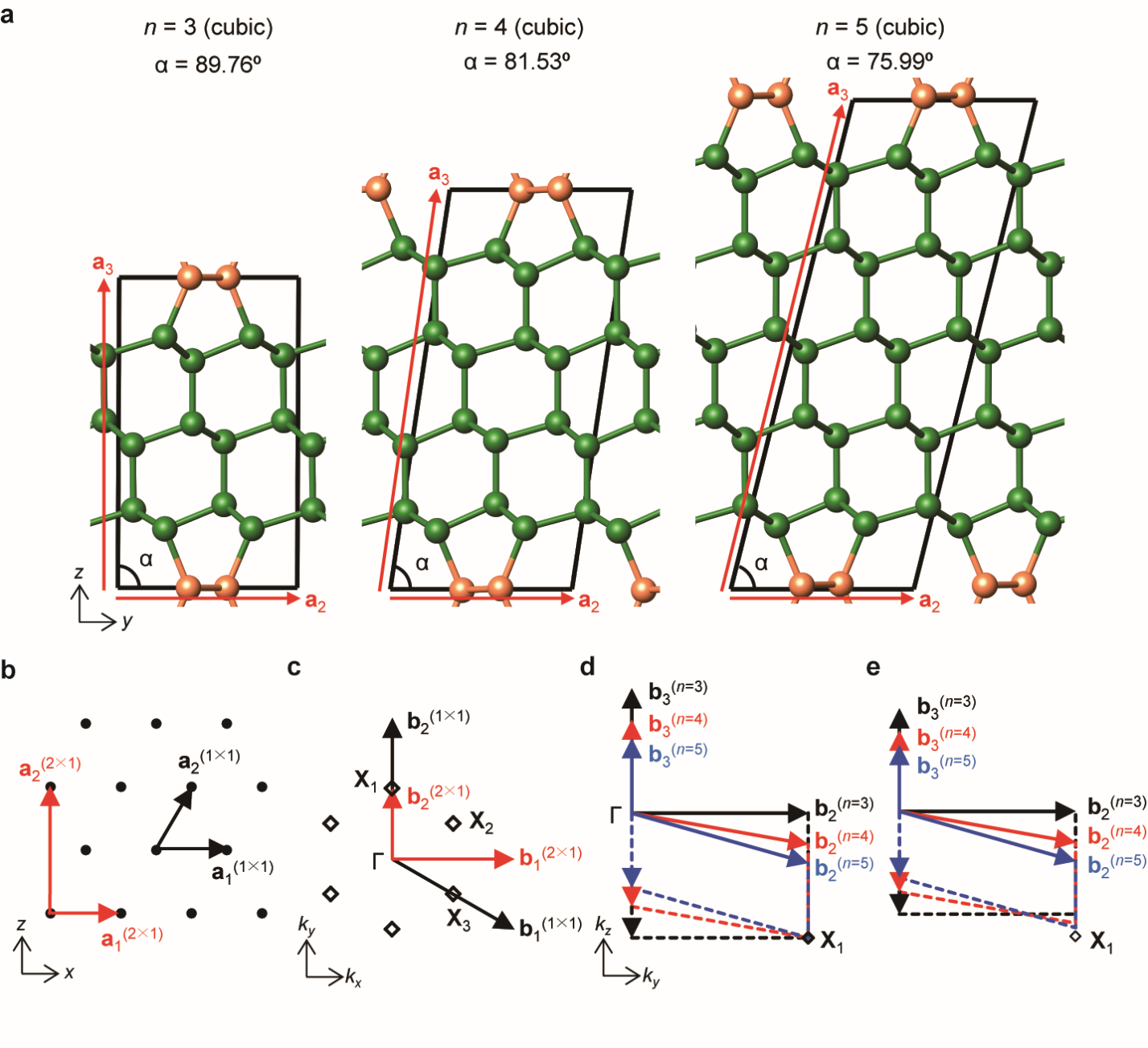


**Supplementary Figure 1.** Illustration of Brillouin zone folding in superlattices. (**a**) Primitive cells and lattice vectors in the Si(111)*_n_*/Si(SC) superlattices (*n* = 3−5) with the cubic-diamond stacking sequence of the Si(111) layers. (**b**) Lattice vectors and (**c**) reciprocal lattice vectors in the 1×1 and 2×1 lateral cells on the two-dimensional hexagonal plane. Projection of the X points in the BZ of *c*-Si onto the *k_x_k_y_* plane is shown. (**d**) Reciprocal lattice vectors in the unit cell of *n* Si(111) layers (*n* = 3−5) in *c*-Si without defective layers, in which the 2×1 lateral cell is chosen. One of the X points (**X**_1_ = **b**_2_ − **b**_3_ in **c**) is folded to the Γ point. (**e**) Reciprocal lattice vectors in the Si(111)*_n_*/Si(SC) superlattices (*n* = 3−5), in which the 2×1 lateral cell is chosen on the (111) plane. The **X**_1_ point (**X**_1_ = **b**_2_ – 1.22**b**_3_) is folded to a **k**-point near the Γ point due to defective layers.


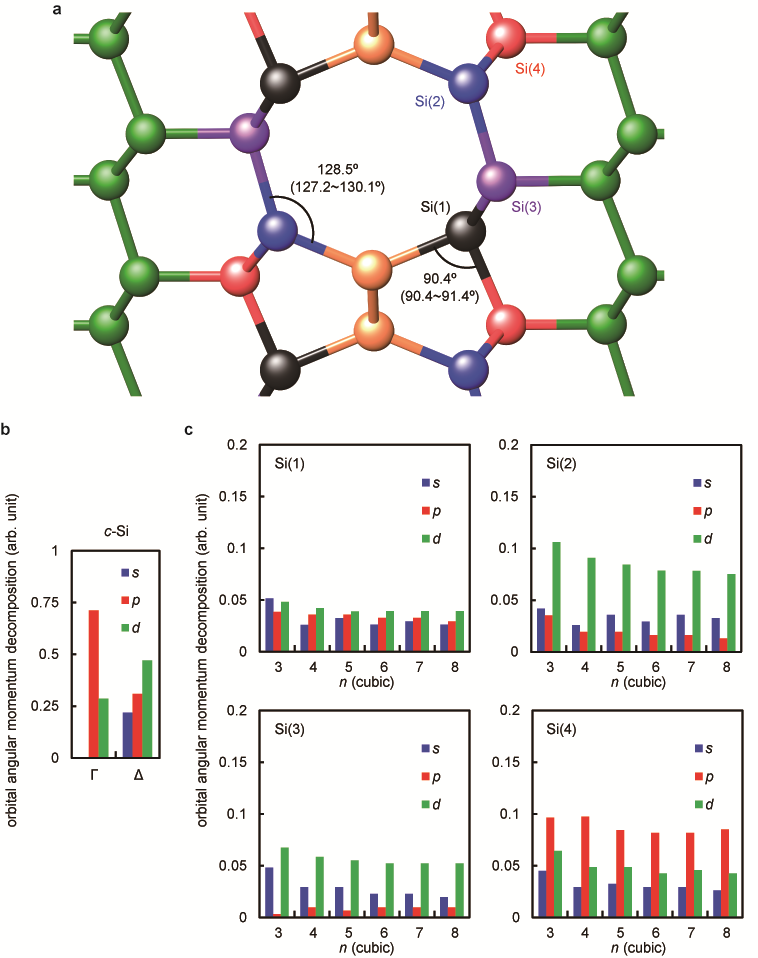


**Supplementary Figure 2.** Orbital characteristics of the lowest conduction band at the Γ point. (**a**) Illustration of the atomic structure near the defective region containing the Seiwatz chains. Colored Si atoms, which are denoted as Si(1), Si(2), Si(3), and Si(4), are located at the interface layers. (**b**) The *s*, *p*, and *d* orbital characteristics of the lowest conduction bands at the Γ and Δ points in *c*-Si. (**c**) In the superlattices with the cubic-diamond stacking sequence of the Si(111) layers (*n* = 3−8), the site-projected *s*, *p*, and *d* orbital characteristics of the lowest conduction band at the Γ point are compared. For each case, the sum of the *s*, *p*, and *d* orbital contributions from all the atoms in the primitive cell is normalized to one.

**Supplementary Table 1.** Space groups and lattice parameters of Si(111)*_n_*/Si(SC) superlattices

| *n* | space group | *a* (Å) | *b* (Å) | *c* (Å) | *α* (°) |
| --- | --- | --- | --- | --- | --- |
| 1 | *Cmcm* (No. 63) | 3.85 | 5.74 | 11.96 | 90.00 |
| Cubic-diamond stacking | | | | | |
| 3 | P2_1_/m (No. 11) | 3.86 | 6.65 | 11.52 | 89.76 |
| 4 | P2_1_/m (No. 11) | 3.86 | 6.66 | 14.82 | 81.53 |
| 5 | P2_1_/m (No. 11) | 3.86 | 6.67 | 18.38 | 75.99 |
| Hexagonal-diamond stacking | | | | | |
| 3 | *P*2_1_/*m* (No. 11) | 3.85 | 6.63 | 11.78 | 79.32 |
| 4 | *Pmm*2 (No. 25) | 3.85 | 6.64 | 14.75 | 90.00 |
| 5 | *P*2_1_/*m* (No. 11) | 3.85 | 6.65 | 18.06 | 82.96 |

**Supplementary Table 2.** Wyckoff positions in Si(111)*_n_*/Si(SC) superlattices

| *n* = 1 | |
| --- | --- |
| 8g (0.25, 0.29370, 0.19519)  4c (0.25, 0, 0.05600) | |
| *n* = 3 with cubic-diamond stacking | *n* = 3 with hexagonal-diamond stacking |
| 2e (0.25, 0.39343, 0.00000)  2e (0.25, 0.26054, 0.18995)  2e (0.25, 0.25466, 0.80908)  2e (0.25, 0.91531, 0.74872)  2e (0.25, 0.58936, 0.26863)  2e (0.25, 0.92081, 0.53949)  2e (0.25, 0.58735, 0.47093) | 2e (0.25, 0.60608, 0.50262)  2e (0.25, 0.57634, 0.02991)  2e (0.25, 0.80575, 0.31459)  2e (0.25, 0.50753, 0.23213)  2e (0.25, 0.00204, 0.75065)  2e (0.25, 0.93314, 0.96070)  2e (0.25, 0.67812, 0.69318) |
| *n* = 4 with cubic-diamond stacking | *n* = 4 with hexagonal-diamond stacking |
| 2e (0.25, 0.39490, 0.99783)  2e (0.25, 0.20839, 0.14613)  2e (0.25, 0.31004, 0.84734)  2e (0.25, 0.98085, 0.80505)  2e (0.25, 0.51129, 0.21267)  2e (0.25, 0.03644, 0.64153)  2e (0.25, 0.45838, 0.37231)  2e (0.25, 0.73004, 0.57825)  2e (0.25, 0.78374, 0.41677) | 1a (0, 0.60819, 0)  1b (0.5, 0.39510, 0)  2e (0, 0.74354, 0.14995)  2e (0, 0.08508, 0.19565)  2e (0, 0.08456, 0.35952)  2e (0, 0.75550, 0.41977)  2f (0.5, 0.25882, 0.14721)  2f (0.5, 0.58581, 0.21088)  2f (0.5, 0.58405, 0.37023)  2f (0.5, 0.24582, 0.41872) |
| *n* = 5 with cubic-diamond stacking | *n* = 5 with hexagonal-diamond stacking |
| 2e (0.25, 0.39470, -0.00129)  2e (0.25, 0.34054, 0.87532)  2e (0.25, 0.02336, 0.83853)  2e (0.25, 0.11628, 0.70345)  2e (0.25, 0.81760, 0.65487)  2e (0.25, 0.90389, 0.52274)  2e (0.25, 0.59982, 0.47827)  2e (0.25, 0.68967, 0.34477)  2e (0.25, 0.38010, 0.30472)  2e (0.25, 0.46840, 0.17390)  2e (0.25, 0.17714, 0.12113) | 2e (0.25, 0.39396, -0.00039)  2e (0.25, 0.21835, 0.12055)  2e (0.25, 0.52761, 0.17341)  2e (0.25, 0.48259, 0.30439)  2e (0.25, 0.13172, 0.34321)  2e (0.25, 0.08841, 0.47624)  2e (0.25, 0.40714, 0.52102)  2e (0.25, 0.36184, 0.65362)  2e (0.25, 0.01580, 0.70419)  2e (0.25, 0.96897, 0.83920)  2e (0.25, 0.29795, 0.87623) |
